# Environmental, biological and cognitive monitoring of children in European primary schools: LEARN crossover study protocol

**DOI:** 10.3389/fpubh.2026.1838168

**Published:** 2026-07-02

**Authors:** Daphne P. Berden, Wai Kei Chu, Ernesto Alfaro-Moreno, Vibeke Heitmann Gutzke, Charikleia Karakosta, Katie Kedwell-Simmering, Varun Kumar, Andreas Massling, Karin Rosenkilde Laursen, Andreas Scope, Carina Veeckman, Rossella Alfano, Jeroen A. J. Vanoirbeek, Tim S. Nawrot

**Affiliations:** 1 Centre for Environmental Sciences, Environmental and Molecular Epidemiology, Hasselt University, Diepenbeek, Belgium; 2 Centre for Environment and Health, Department of Public Health and Primary Care, KU Leuven, Leuven, Belgium; 3 International Iberian Nanotechnology Laboratory, Braga, Portugal; 4 Department of Public Health, Aarhus University, Aarhus, Denmark; 5 Envirometrics Technical Consultants SA, Chalandri, Greece; 6 Advanced Technology, MANN+HUMMEL GmbH, Ludwigsburg, Germany; 7 Department of Environmental Science, Aarhus University, Roskilde, Denmark; 8 imec-SMIT-Vrije Universiteit Brussel, Brussels, Belgium

**Keywords:** air remediation, biomonitoring, cognitive performance, crossover study design, indoor air pollutants, school-aged children, study protocol

## Abstract

Poor indoor air quality (IAQ) in schools is of particular concern as it can negatively impact children’s cognitive development, health, and well-being. Measuring IAQ-related parameters, assessing children’s internal and external exposures, and developing solutions for reducing exposure are therefore critical to protecting children’s health. The use of air purifiers can be a good mitigation strategy to improve IAQ in classrooms, but their association with health effects and cognitive outcomes in children remains unclear. The LEARN crossover study protocol aims to address this gap by evaluating the impact of a novel air purification intervention on IAQ, biomarkers of exposure, clinical health parameters, and cognitive performance in primary school children (*n* = 450). In this single-blinded (teachers/students blinded) crossover study conducted in primary school classrooms in Belgium, Denmark, and Greece, the participating classrooms are cluster-randomized via a computer-generated sequence to the order of receiving the intervention and the sham intervention (air purifiers operated with or without filters), with a washout period between study phases. Active and passive measurements of volatile organic compounds (VOCs) and aldehydes, active measurements of polycyclic aromatic hydrocarbons (PAHs), as well as monitoring of particles (PM_10_, PM_2.5_, ultrafine particles, black carbon) and microclimate parameters are performed. At pre-defined timepoints, urine samples, blood samples, and skin swabs are collected and assessed for exposure biomarkers and health parameters. To ensure cross-site comparability, children’s cognitive performance is evaluated using Danish, Greek, and Dutch versions of standardized tests: the Continuous Performance Test, Symbol Digit Modalities Test, Memory Span Task, STROOP Task, and Signal Detection Test. Additional health assessments include anthropometric and blood pressure measurements, and psychological well-being questionnaires. Statistical analyses include mixed models to explore exposure-response relationships between children’s external and internal exposures, health, and cognitive outcomes. This protocol presents a comprehensive approach to assess indoor air exposures and the impact on children’s health and cognition, as well as the effectiveness of the air purification strategy. The results of this study can provide a reference for novel IAQ remediation strategies in schools and help promote the application of human biomonitoring in risk assessment of children’s indoor exposure.

**Clinical trial registration:**

https://clinicaltrials.gov/study/NCT06197477, Identifier NCT06197477.

## 1 Introduction

According to the 2025 OECD countries (Organization for Economic Co-operation and Development) report on education, primary school children spend on average 804 h per year in the classroom, which makes approximately 31% of their waking hours on school days (1). These classrooms are characterized by substantially higher occupancy densities compared to most other building types, sometimes reaching density levels up to four times greater than those observed in typical office environments (2). Despite school efforts to improve indoor air quality (IAQ), students are still frequently exposed to suboptimal conditions (3). Exposure to poor IAQ has been shown to significantly impact children’s health, comfort, and academic performance. Moreover, prolonged exposure to indoor air pollution can contribute to cardiovascular and respiratory diseases, adversely affect the central nervous system, and result in cognitive impairment and developmental delays (4–12). Children are particularly vulnerable due to their developing organs and physiological systems, higher breathing rates, and behaviors that increase their exposure (7). Ultimately, managing IAQ in educational facilities is crucial, as it directly affects their health, academic performance, and attendance (13).

Volatile organic compounds (VOCs), semi-volatile organic compounds (SVOCs), and ultrafine particles (UFPs) are the indoor air pollutants of major concern due to their potential impacts on children’s health and development (4, 9, 14–16). Current strategies to improve IAQ in schools, such as opening windows for ventilation, help dilute indoor pollutant concentrations with outdoor air. However, this approach only works in areas with clean outdoor air and is problematic for schools located near busy roads (4). In such cases, pollutants such as combustion by-products, ozone (O₃), and allergens, among many others, can enter the classroom (17). Additionally, natural ventilation is not a viable option in unfavorable weather conditions. In these situations, using an air purifier can be an effective mitigation strategy to improve classroom IAQ (18–21).

Current epidemiological evidence on the association between air purifier use and health outcomes remains limited and inconsistent, especially regarding the primary school setting (18). Although recent studies indicate that filtration-based systems can improve IAQ as well as respiratory health, cardiovascular parameters, and academic performance, substantial gaps in the evidence persist (5, 18, 22). These gaps arise from a shortage of air quality intervention studies in primary school settings, coupled with highly variable regional exposure levels. To overcome this, future research must establish a more globally diverse framework that incorporates real-time data across varying exposure levels, allowing for a comprehensive evaluation of the overall health effects in this vulnerable population.

The use of human biomonitoring to assess internal exposure and early biological effects of environmental chemicals also remains underutilized, particularly in vulnerable populations such as children, which is identified as one of the priority areas in the European Exposure Science Strategy (23, 24). Environmental monitoring measures only ambient concentrations, whereas biomonitoring accounts for individual variations in inhalation rates, physical activity, and metabolism. By quantifying the actual internal doses, biomonitoring directly links environmental exposure to physiological uptake, providing more precise exposure metrics to refine exposure-response relationships (25). Consequently, there is a clear need for integrated studies that combine environmental monitoring, biomonitoring, and cognitive assessments within real-world intervention frameworks across multiple European school settings.

The European Union-funded project, LEARN (deveLopment of novEl Assessments for indoor aiR quality moNitoring and impact on children’s health), aims to better understand IAQ in schools and investigate its impact on children’s cognitive performance and general health, and evaluate the efficacy of air purification. To achieve this, the study introduces an integrated, multi-country trial across Belgium, Denmark, and Greece. The study’s primary novelty lies in its paired framework, which links classroom air quality data, including concentrations and profiles of indoor and outdoor air pollutants and microclimate parameters, with advanced human biomonitoring to track internal pollutant exposure alongside children’s health outcomes. Furthermore, the intervention utilizes a novel air purification system engineered and customized by MANN+HUMMEL specifically for this study. By evaluating this innovative technology across distinct geographic regions, LEARN will directly link environmental exposure to physiological uptake, providing a robust framework to optimize children’s health and cognition in educational environments and to investigate the efficiency of air purification.

## 2 Study design

Here, we describe the study protocol of a single-blinded (teachers/students blinded) crossover intervention study conducted as part of the LEARN project. The study aims to evaluate IAQ in schools, the level of exposure, and its effects on the health and well-being (cognition, blood pressure, quality of life) of school children aged 9 to 12 years old. Additionally, this study explores improved approaches for characterizing IAQ and evaluating intervention effectiveness. A crossover design was selected to allow each participant to serve as their own control, thereby reducing inter-individual variability and increasing statistical power in detecting short-term effects of IAQ interventions.

### 2.1 Study setting and eligibility criteria

LEARN is a multicenter study with case study sites in three European countries: Belgium, Denmark, and Greece. The Belgian study population is recruited from the Flemish Region of Belgium, in the Flemish provinces of Limburg, Antwerp, and Flemish Brabant. For the Danish and Greek sites, recruitment focuses on primary schools in the municipality of Aarhus and in Thessaloniki, respectively.

According to geographical location (feasibility, deprivation, etc.), schools are contacted (mail, phone, video call) for participation. The principal completes a questionnaire providing a detailed description of the school’s infrastructure. Schools with an air filtration system and/or policies requiring windows to remain closed are excluded. Sufficient space should also be present in the classrooms to position the filtration devices optimally, with at least 30 cm of clearance around them, to ensure effective intervention. For reasons of generalizability and external validity, we intentionally avoid imposing additional restrictive exclusion criteria. This approach ensures that the study remains representative of the real-life school environments where such interventions would be implemented.

Any primary school located in the included regions of Belgium, Denmark, or Greece complying with these criteria can participate, provided that at least two classes from the same school from the 4th, 5th, and/or 6th grade agree to take part in the study. Once the classes are confirmed, an information session is organized with the principal, teachers, parents, and students. During this session, the study is explained to the students in an age-appropriate manner. Additionally, each child receives an envelope containing detailed information about the study and a consent form for their legal guardian(s) to sign. Insufficient knowledge of the Dutch, Danish, or Greek language by the child or the child’s guardian(s) leads to exclusion from the study, due to the availability of study materials limited to these languages.

### 2.2 Sample size calculation

A linear mixed-effects model will be applied to evaluate the association between the IAQ, the intervention, and the measured clinical parameters and biological biomarkers. Random intercepts will be specified for school, class, and child, and site-specific estimates will be combined through meta-analysis. As no pilot data were available across all sites, a simulation-based power analysis was conducted in R. The simulations assumed a within-person residual standard deviation (SD) of 1, and intra-class correlations (ICCs) of 0.05 for school, 0.10 for class, and 0.50 for child. For each scenario, 1,000 datasets were generated. In each dataset, the group × time contrast at the final time point was estimated using a linear mixed model, and site-specific results were combined via random-effects meta-analysis. The proportion of simulations yielding a two-sided *p* < 0.05 was recorded. Results indicated that recruiting approximately 150 children per country would provide 90% power to detect a medium-sized standardized intervention effect (Cohen’s d = 0.5 SD). This medium effect was selected as a pragmatic benchmark and should not be interpreted as the minimum clinically important difference for each outcome. We acknowledge that smaller effects than d = 0.5 SD may still be clinically or public-health relevant, but our study may have limited power to detect such smaller effects, and null findings should therefore be interpreted with caution.

### 2.3 Sampling timeline

**Figure 1:**
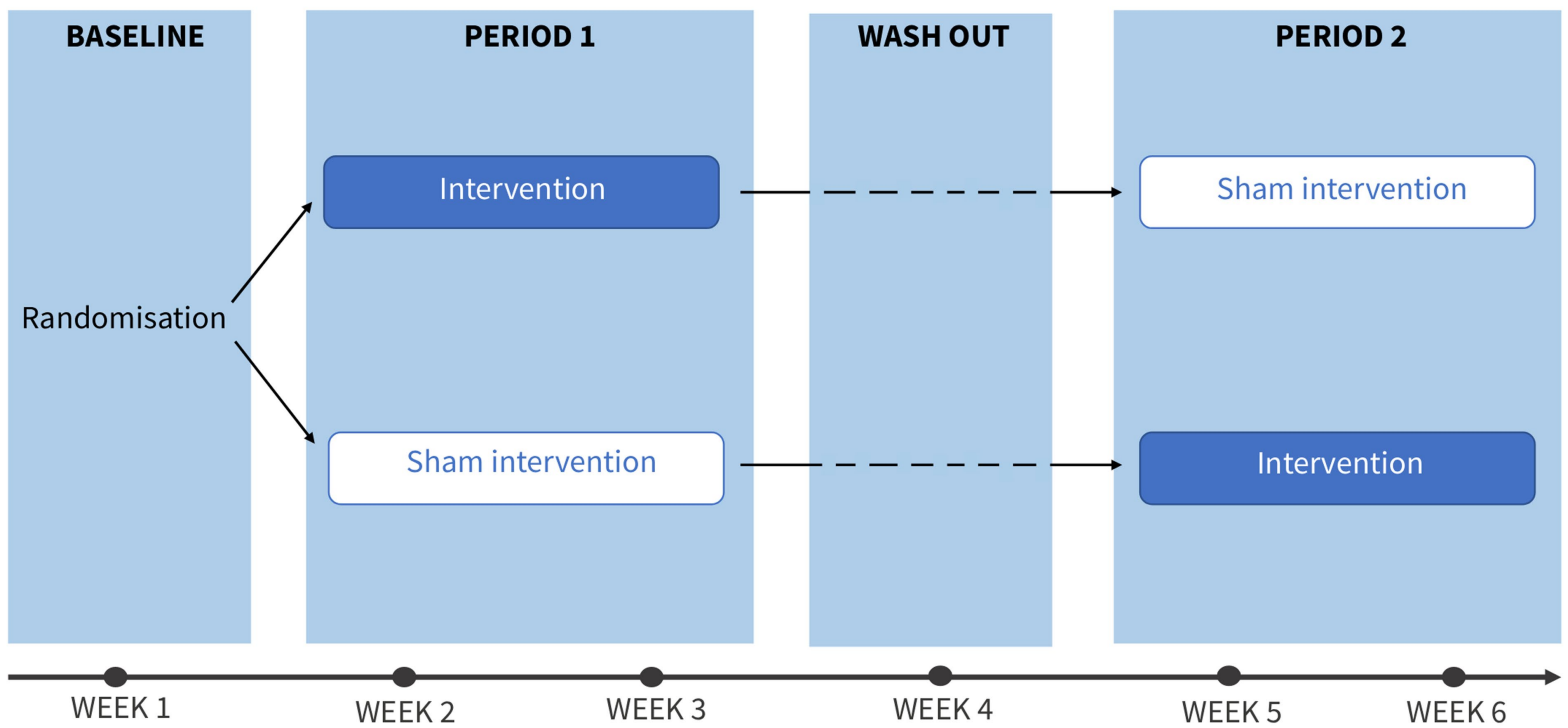
Schematic diagram of the single-blinded crossover study design in the LEARN study.

The LEARN study consists of four phases and takes approximately 6 to 7 weeks to complete for each school (Figure 1).

1. Baseline Period (BaP): No intervention is implemented at baseline (1 week).
  The participating classes are randomly assigned to one of two sequences in the crossover design: either starting with the intervention phase (installation of air purification units with filters) followed by the control phase (sham-intervention with installation of air purification units without filters)(R1), or vice versa (R2). Randomization is performed at the class level using computer-generated random numbers. The study is single-blinded, hence participants and teachers are unaware of the assignment of the intervention phase.
2. Intervention Period 1 (IP1): The assigned (sham-) intervention is implemented and administered over 2 weeks.
3. Washout Period (WP): A washout period of 1 to 2 weeks follows IP1, ensuring that the acute environmental and physiological effects of the initial intervention are fully diminished to maintain a true crossover design (26–29). Furthermore, this timeframe accommodates the academic calendar and classroom scheduling constraints. Potential carry-over effects will be further evaluated and adjusted for in the statistical analysis.
4. Intervention Period 2 (IP2): The intervention status switches between the two groups, and the second phase of the study is conducted.

Throughout the various study phases, data collection is divided into exposure assessment (detailed in Section 3) and health outcome measurements (detailed in Section 4). These activities evaluate children’s external and internal exposure to selected pollutants, alongside their cognitive function, clinical parameters, and skin microbiome.

### 2.4 Intervention

**Figure 2:**
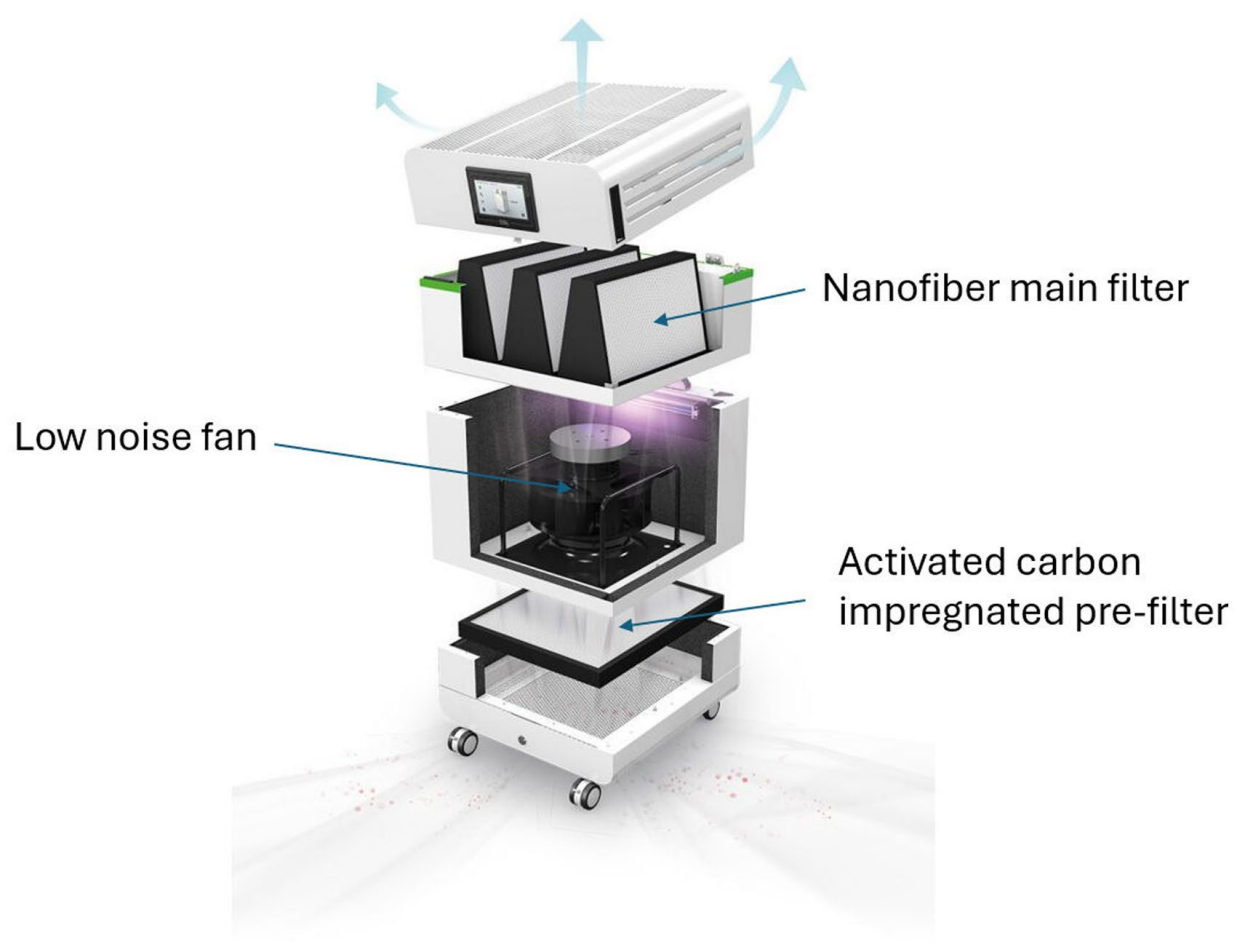
Schematic overview of the OurAir SQ 1750 mobile air purification system.

As an intervention, we use the OurAir SQ 1750 Mobile Air Purification System for Virus-Free Air Indoors, engineered and customized by MANN+HUMMEL (Figure 2). The main filter stage is equipped with a high-efficiency nanofiber filter media, combining high separation efficiency (> 90% at 0.3 μm; equivalent to ePM_1_ 90% according to ISO 16890) with low differential pressure. The prefilter provides removal of coarse and fine particulate matter (e.g., dust, hair, pollen) and is additionally equipped with activated carbon layers selected for effective adsorption of VOCs (specified target gases: n-butane, toluene).

Prior to the study, a simulation was performed to determine the optimal placement and operating point of the air purifier in the classroom. The optimum was defined as an air-cleaning rate corresponding to six air changes per hour (ACH). This 6-ACH target is applied by adjusting the device setting to the specific volume of each participating classroom. For the initial pilot study, however, the device was deliberately operated at the maximum airflow setting (1885.9 m^3^/h) to assess performance under worst-case conditions; this setting still resulted in a tolerable noise level (45.7 dB(A)).

To ensure effective blinding, the sham-intervention classroom has a mobile air purifier with the main filter and activated carbon prefilter removed. This device mirrors the operating noise of the functional units in the intervention classroom, neutralizing sound as a potential confounding variable (30).

## 3 Exposure assessments

The indoor exposome (referring to the cumulative measure of external and internal exposures within the school environment) is characterized via screening questionnaires, indoor environmental monitoring, and biological monitoring.

### 3.1 External exposure

#### 3.1.1 Classroom and building characteristics and classroom activities

A screening questionnaire developed by the World Health Organization (WHO) (31), which addresses the structural and geographical characteristics of the school building and participating classrooms, is completed by the school’s responsible before the sampling campaign. The questionnaire examined (1) school building and classroom characteristics, (2) potential sources of indoor chemicals, and (3) other factors that affect IAQ (e.g., ventilation types and cleaning activities). More specifically, following questions are included: location and year of construction of the school; classroom orientation and dimension; type of surrounding environment (urban, suburban, rural), number of children and teachers (occupancy density), building heating system (electricity, fuel), building envelope (materials used to make floors, walls, windows, furniture), ventilation type (manual, mechanical, or hybrid), and visual inspection for mold, pests and dampness. Also, an overview of classroom activities during the sampling period, including the absence of children in the classroom (due to physical education) and the potential use of chemicals (e.g., painting), is provided through informal conversations with the head teacher of each participating class.

The data will be used to evaluate potential sources of indoor air pollutants and assess comparability between the classrooms participating in the study. The characteristics of school buildings, outdoor pollution sources, and the behavior of building occupants (activities in classrooms, cleaning routines, etc.) are expected to play a key role in understanding the IAQ of schools, as it depends strongly on the interaction between the building and the outdoor environment, and on the way the building is used, including the behavior of occupants (32).

#### 3.1.2 Indoor environmental monitoring

The selection of indoor air pollutants investigated in this study takes into account the following factors: (1) Current list and wish list of priority substances included in a screening tool developed by the WHO for assessment of health risks from combined exposure to multiple chemicals in indoor air in public settings for children (33); (2) List of indoor air pollutants previously investigated in EU projects on IAQ in primary schools in Europe (e.g., SINPHONIE, InAirQ) (32, 34, 35); (3) Findings from a systematic review of human biomonitoring studies of children’s exposure to indoor air pollutants in school and home environments (25); (4) Other indoor pollutants investigated in European primary schools in peer-reviewed journals (36–39).

VOCs, aldehydes, polycyclic aromatic hydrocarbons (PAH), and particles are selected as target pollutant groups. Table 1 shows the individual target pollutants selected for indoor air measurements in the LEARN study. The current list of indoor pollutants is a provisional selection at this stage, and will be revised based on the results of subsequent measurement campaigns of LEARN. The study also selected IAQ-related microclimate parameters, as shown in Table 2, for measurement based on international standards such as the ISO 7726 (40) and the European standard EN 16798–1 (41), as well as the previous EU research projects (32, 34).

**Table 1:** List of pollutants selected for indoor air measurements in the LEARN study.

Selected air pollutants classified by chemical groups	
VOCs	Benzene
	Toluene
	Ethylbenzene
	o-Xylene
	m + p-Xylene
	Trichloroethylene
	Tetrachloroethylene
	Styrene
	1,4-dichlorobenzene
	Acetone
	Tetrachloromethane (Carbon tetrachloride)
	2-methylbutane
	n-butyl acetate
	n-decane
	1,2,3-trimethylbenzene
	Ethanol
	Acetonitrile
	2-methylpentane
	3-methylpentane
	Methyl cyclopentane
	2-methylhexane
	2,3-dimethylpentane
	3-methylhexane
	n-Heptane
	n-Hexane
	n-Pentane
VOCs (Terpenes)	Limonene
Aldehydes	Formaldehyde
	Acetaldehyde
	Acrolein
Particles	PM_2.5_
	PM_10_
	Ultrafine particles (UFPs)
	Black carbon (BC)
PAHs	Naphthalene (Naph)
	Benzo(a)pyrene (B[a]P)
	Phenanthrene (Phe)
	Benz[a]anthracene (B[a]A)
	Benzo[b]fluoranthene (B[b + j]F)
	Benzo[k]fluoranthene (B[k]F)
	Dibenz[a,h]anthracene (D[a,h]A)
	Indeno[1,2,3-cd]pyrene (Inp)
	Acenaphthene (Ace)
	Acenaphthylene (Acy)
	Anthracene (Ant)
	Benzo[g,h,i]perylene (B[ghi]P)
	Chrysene (Chry)
	Fluoranthene (Fln)
	Fluorene (Flu)
	Pyrene (Pyr)

**Table 2:** List of microclimate parameters selected for indoor measurements in the LEARN study.

Microclimate parameters
Carbon dioxide (ppm)
Indoor airflow velocity (m/s)
Relative Humidity (%RH)
Dry-bulb temperature (°C)
Natural wet-bulb temperature (°C)
Globe temperature (°C)

**Figure 3:**
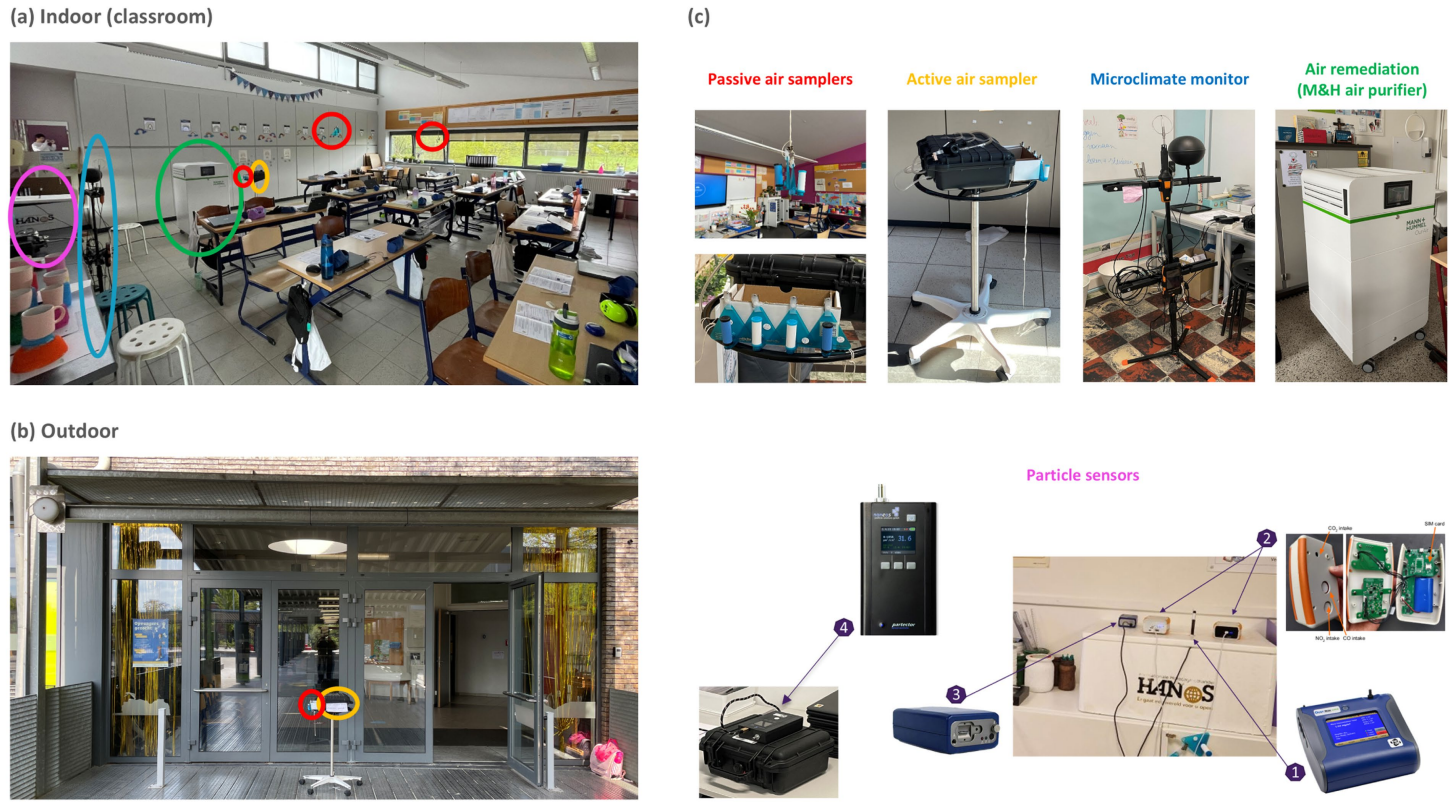
Overview of indoor (classrooms) and outdoor sampling settings in Belgium. **(a)** Placement of sampling devices inside the classroom and **(b)** at an outdoor location. **(c)** Detailed view of the devices: Passive samplers (red): Radiello diffusive samplers to measure VOCs (white, RAD130) and aldehyde (blue, RAD165) concentrations. Active samplers (yellow): Pumps connected with target-specific sorbent media. Microclimate monitor (blue): Testo 400 connected with the Wet Bulb Globe Temperature (WBGT) set. Air remediation (green): Mann+Hummel OurAir SQ 1750 Mobile Air Purification System. Particle sensors (purple): (1) TSI DustTrak™ DRX (Model 8,533) for simultaneous PM measurements, (2) Customized multi-sensor nodes for particle measurement, (3) AethLabs microAeth® (Model AE51) for black carbon (BC) measurement, (4) Naneos Partector for ultrafine particles (UFPs) measurement.

The environmental monitoring strategies of pollutants used in the LEARN study, including duration, period, and sampling location, parallel outdoor measurements, are developed mainly based on the methods recommended in the ISO 16000 series and the European standards for ambient air, as well as sampling methods adopted by WHO, NIOSH, previous EU projects, and peer-reviewed journals (32, 34, 42–44). There are two methods used to sample chemical pollutants: passive diffuse air sampling and active, pump-driven air sampling. An overview of all indoor (classroom) and outdoor sampling locations is shown in Figure 3.

This environmental monitoring plan is primarily designed for and applied to the Belgian sites. When sampling campaigns are extended to the Danish and Greek sites, the plan will be adapted to local conditions. These adaptations are necessary to accommodate the variations in school schedules and specific spatial or logistical constraints in Denmark and Greece. However, to ensure comparability between countries, the key parameters and procedures across all monitoring sites will be standardized. Specifically, the target pollutants and parameters (Tables 1, 2) and the use of sampling approaches will be identical. In addition, all air samples collected from all sites will be transported to and analyzed by the laboratory at KU Leuven using standardized internationally recognized protocols.

Ideally, local site adaptations will be restricted to the physical placement of samplers and sampling times to align with local school hours. However, if specific samplings cannot eventually be performed at certain sites due to local logistical constraints, the pooled analyses will utilize only overlapping standardized environmental parameters collected from all sites. Any non-overlapping measurements will be analyzed independently as high-resolution datasets for specific sites to provide deeper mechanistic insights.

#### 3.1.3 Active air sampling and analysis

**Figure 4:**
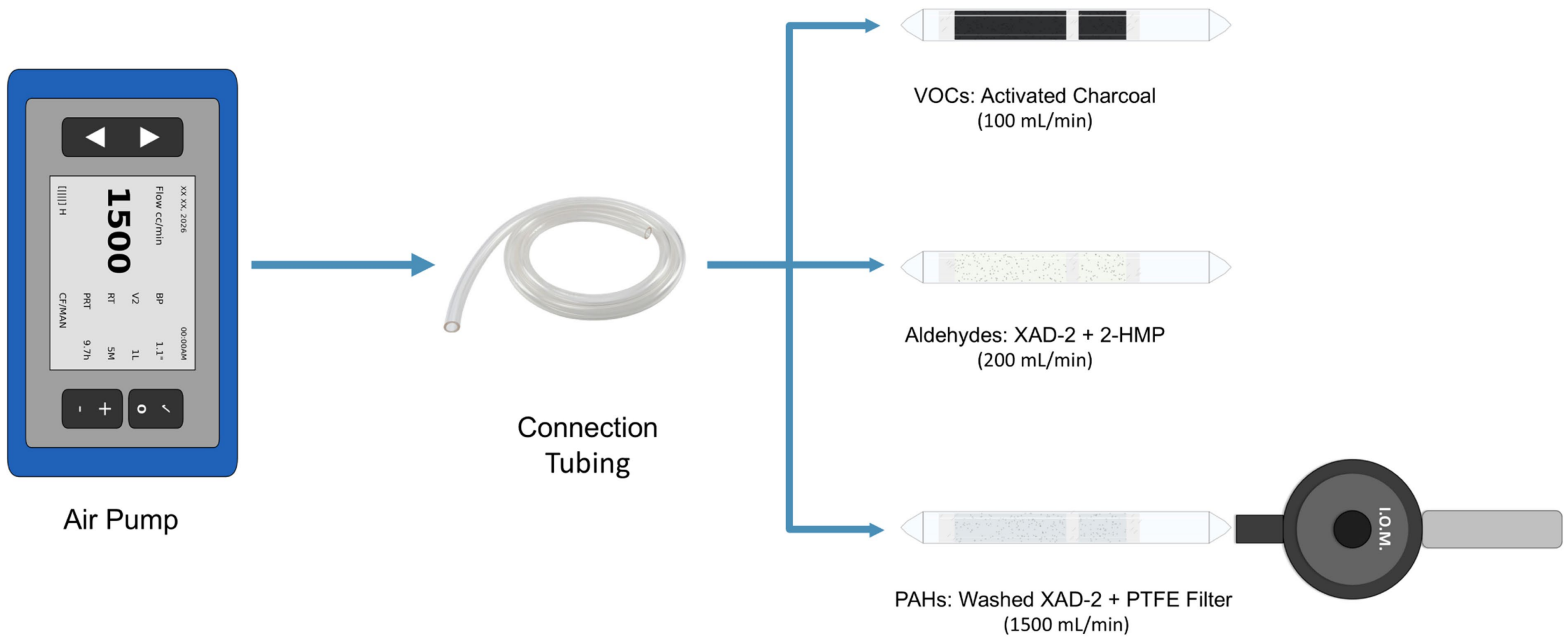
Schematic diagram of the setting of active air samplers for VOCs, aldehydes, and PAHs. It consists of a pump connected to a pollutant-specific sorbent tube. For VOCs, an activated charcoal tube is used at 100 mL/min. For aldehydes, a XAD-2 sorbent tube doped with 2-(hydroxymethyl) piperidine (2-HMP) is used at 200 mL/min. For PAHs, the setup includes a PTFE filter upstream of a washed XAD-2 sorbent tube at 1500 mL/min.

Active air sampling is performed at one location in each classroom and one location outside the school building (e.g., playground, but under a protective roof) during school hours on regular school days throughout the week (2 days during BaP, 3 days during IP1 and IP2). Air samples are collected using an air sampling pump (1–5,000 mL/min) connected with corresponding sorbent tubes (for VOCs, aldehydes, and a gaseous fraction of PAHs) and PTFE filters (for a particulate fraction of PAHs), as visualized in Figure 4. VOCs are collected on activated charcoal tubes at a flow rate of 100 mL/min according to EN 14662–2; aldehydes on XAD-2 tubes coated with 2-(hydroxymethyl)piperidine (200 mL/min; NIOSH 2539); and PAHs on a combined assembly of PTFE filters and washed XAD-2 tubes (1,500 mL/min; NIOSH 5515). The air samples are then desorbed by solvent and analyzed by gas chromatography (GC), with flame ionization detection (FID) for VOCs and aldehydes, and with mass spectrometry (MS) for PAHs (45, 46).

During the sampling campaign at each school, field blank samples and laboratory blank samples are collected to control contamination during transportation and sampling. All pumps used for active sampling are previously calibrated and verified after each sampling. After each sampling, the sorbent tubes and filters are capped and sealed in a clean, hermetically closed container and transported to the laboratory for refrigeration at −20 °C.

#### 3.1.4 Passive air sampling and analysis

**Figure 5:**
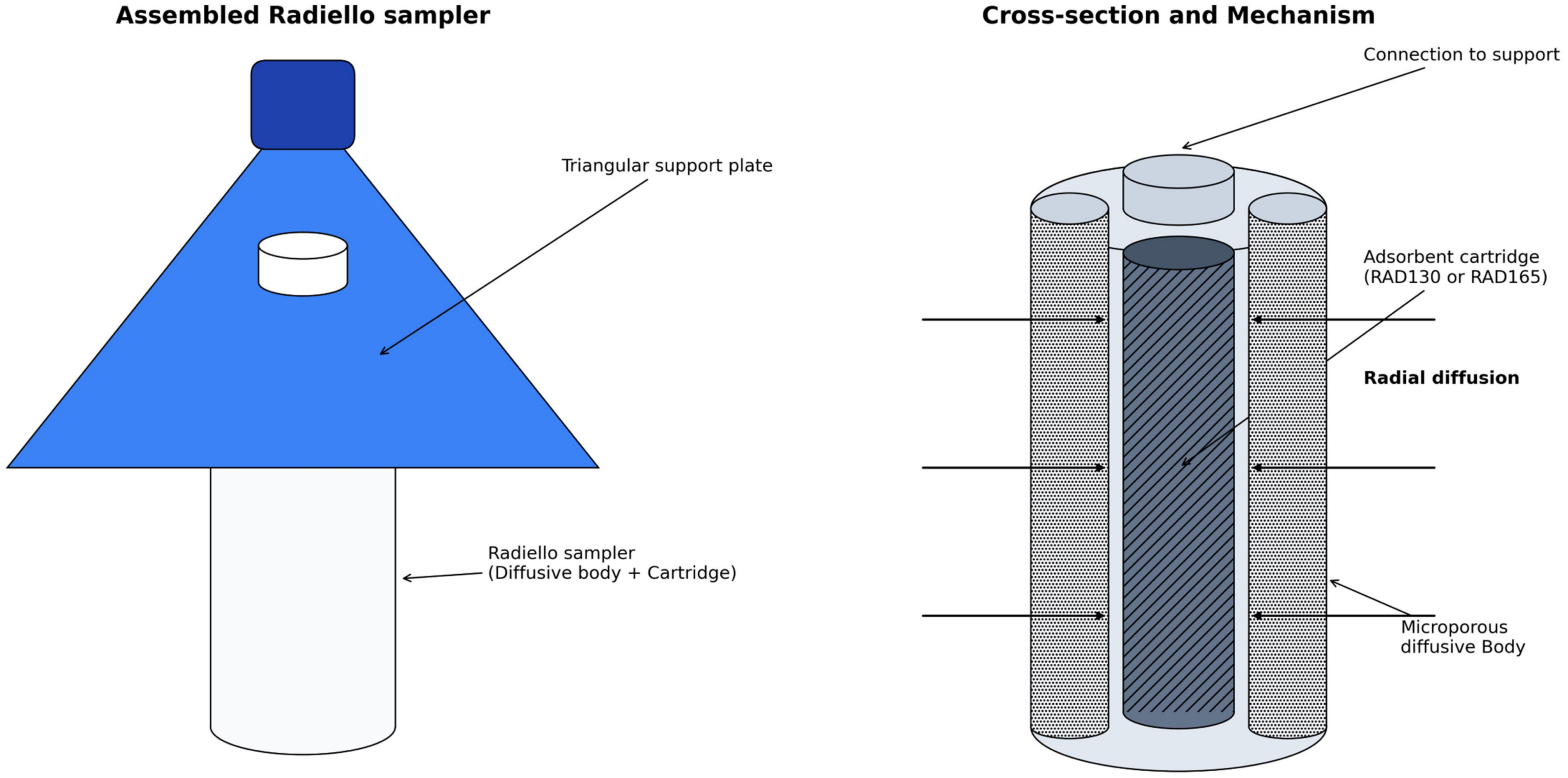
Schematic diagram of a passive air sampling setup. (Left) The assembled Radiello diffusion sampler consists of a triangular support plate and a diffusive body. (Right) The cross-section shows the diffusion mechanism. The pollutant-specific adsorbent cartridge is placed inside the diffusive body. VOCs are sampled with RAD130 and analyzed according to EN 14662–5. Aldehydes are sampled with RAD165 and analyzed according to ISO 16000-4 (47).

Passive air sampling is conducted over 5 days (from Monday morning to Friday afternoon). According to the ISO 16000-5 and ISO 16000-4 methods, respectively, using Radiello diffusive samplers (Figure 5; RAD130 for VOCs, RAD 165 for aldehydes) to collect air samples at three to four locations in each classroom and one location outside the school building (e.g., playground) (47). During the weekend of a specific “Intervention Period,” the cartridges are kept sealed in a clean glass tube from Friday after school hours until Monday morning when the school hours start again. For IP2, a new set of diffusive samplers is used. VOCs are determined by solvent desorption and GC-FID according to EN 14662–5. Aldehydes are determined by solvent desorption and high-performance liquid chromatography (HPLC) equipped with an ultraviolet (UV) absorption detector operating at 360 nm according to ISO 16000-4.

During the sampling campaign at each school, duplicate samples are collected as internal quality control, while field blanks and laboratory blanks are collected to control contamination during transportation and sampling. All samples are sealed and refrigerated at −20 °C after sampling.

#### 3.1.5 Particle measurements

Particle measurements are performed on-site in both R1 and R2 during BaP, IP1, and IP2 to measure mass and number concentrations of indoor airborne particles. The particle number concentration is measured by using Naneos Partector 2 (number concentration range: from 0–10^6^ cm^−3^; average particle diameter range: from 10 to 300 nm) (48). PM_Total_, PM_1_, PM_2.5_, and PM_10_ concentrations are measured using portable mid-cost sensors, i.e., Metone Aerocet 532 particle monitor for measurements in Denmark and DustTrak DRX Model 8,533 (Aerosol concentration range: 0.001 to 150 mg/m^3^; particle size range: 0.1 to 15 μm) for measurements in Belgium. To ensure data redundancy, customized multi-sensor nodes are co-located with the DustTrak unit. Black carbon (BC) levels are determined using a MicroAeth AE51 aethalometer (concentration range: 0–1 mg BC/m^3^) or an Aethlabs MA-200 black carbon monitor. These devices were based on previous literature, and the sampling strategy is aligned with another EU project (InChildHealth: https://inchildhealth.eu/) to obtain standardized data on pollutants for future comparisons. Indoor air pollution levels at the participants’ residences will also be determined using Geographical Information Systems (GIS). Inter-device comparability and calibration are implemented. Before the field campaigns, to eliminate potential systematic measurement differences between sensors from different brands, all devices are co-located in a controlled environment before deployment, ensuring harmonized and comparable particle concentration data across cohorts. Annual calibration checks are also performed by the supplier.

#### 3.1.6 Microclimate monitoring

**Figure 6:**
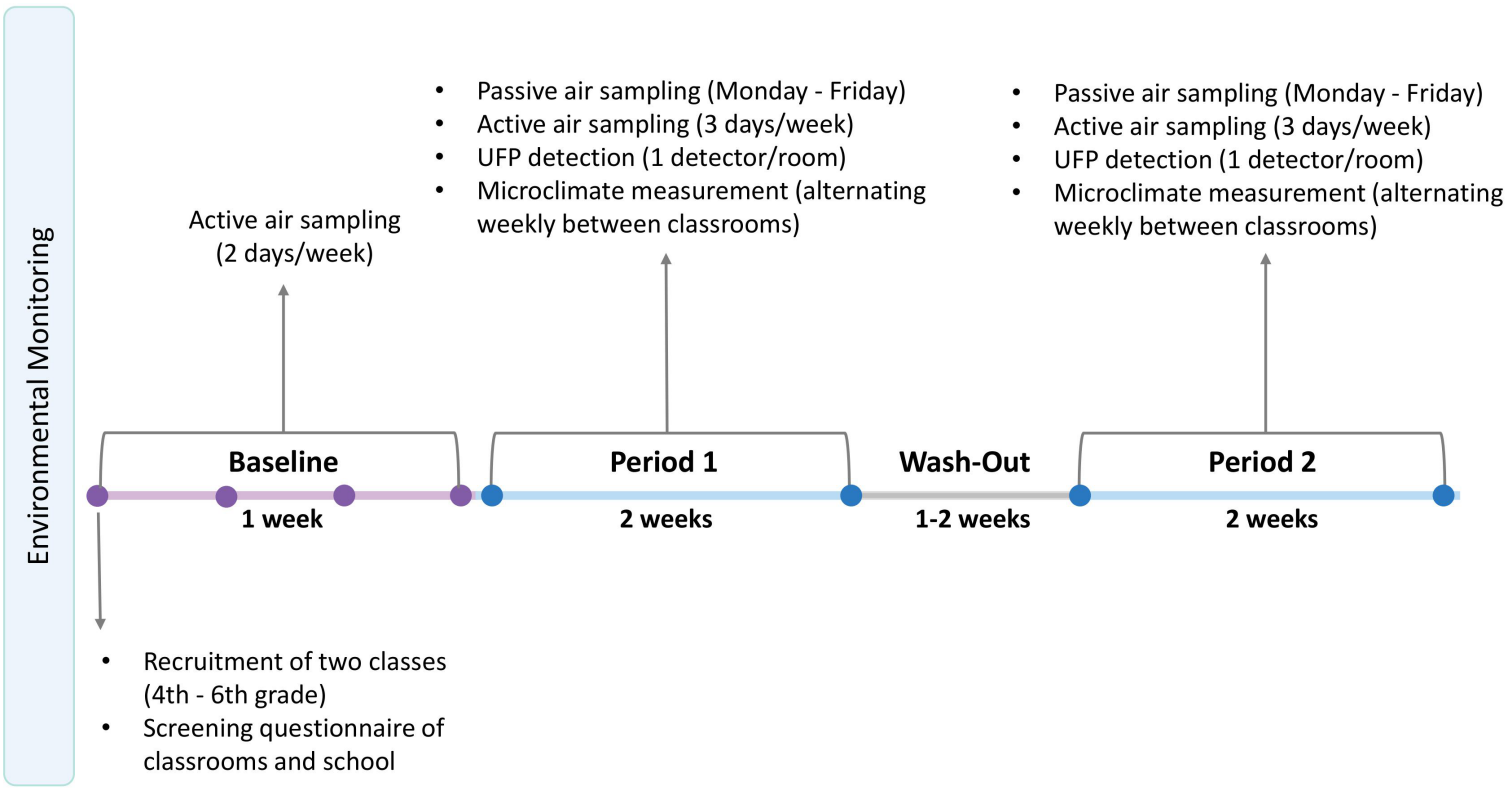
Schematic diagram of environmental monitoring.

During IP1 and IP2, selected IAQ-related microclimate parameters, including carbon dioxide (CO₂) concentration (an indicator of ventilation rate), indoor airflow velocity, relative humidity, dry-bulb temperature, natural wet-bulb temperature, and globe temperature, are monitored continuously for five school days per week, alternating between R1 and R2 each week, as shown in Figure 6. The measurements are conducted using a universal IAQ instrument (model Testo 400, Testo SE & Co. KGaA) equipped with the IAQ and comfort kit (0563 0401) and the Wet-bulb Globe Temperature (WBGT) Kit (0618 7,220). All measurements are performed according to ISO 7726 standards. The WBGT index was calculated based on the ISO 7243 standard, which is a heat stress index and represents the thermal environment to which an individual is exposed.

### 3.2 Biomonitoring of internal exposure

#### 3.2.1 Blood sampling

**Figure 7:**
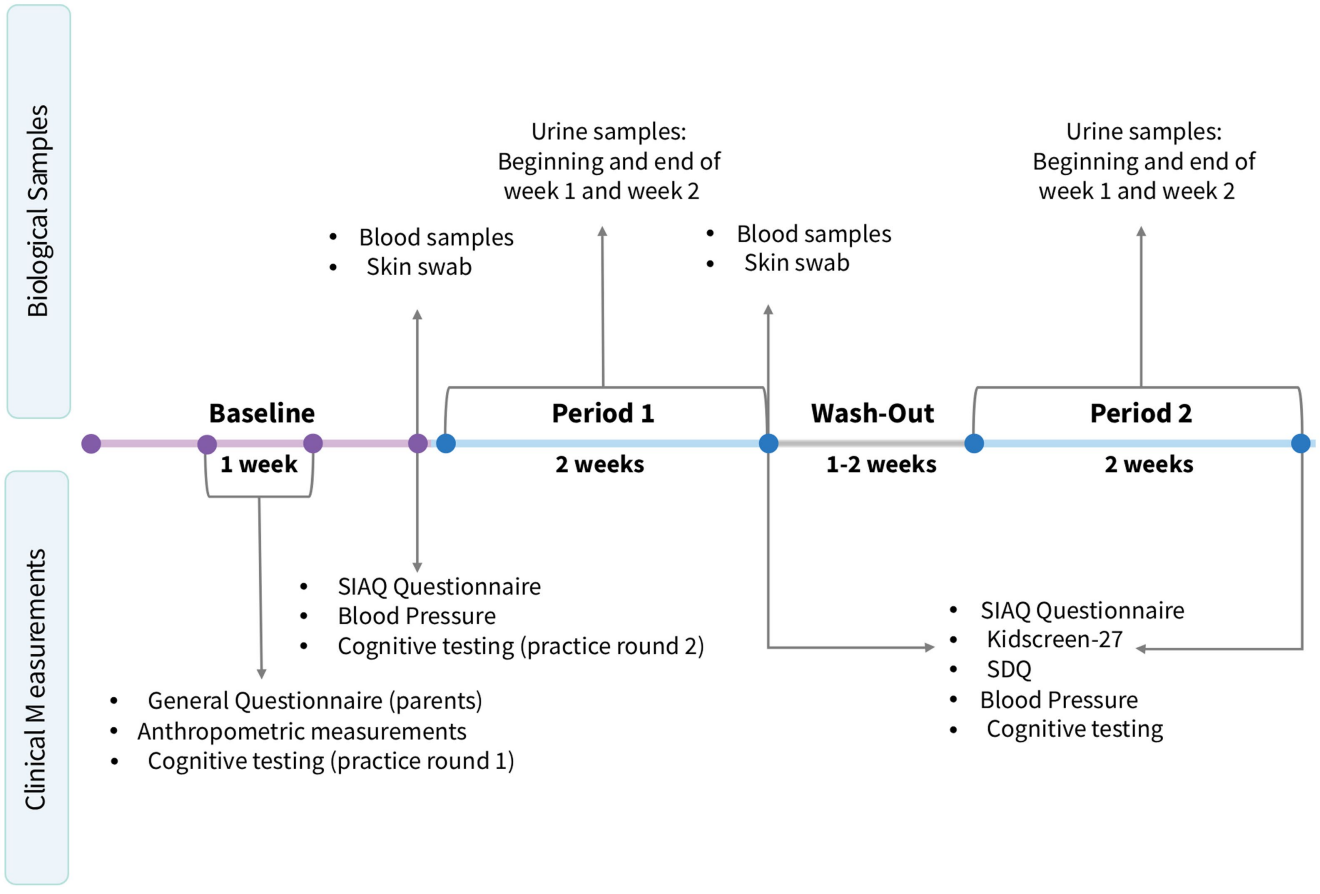
Schematic diagram of clinical measurements and biological monitoring.

Blood samples are collected from Belgian participants who have provided additional informed consent. To ensure standardized results, collection occurs between 12:00 and 14:00 on the final day of both BaP and IP1 (Figure 7). The blood samples will be analyzed for complete blood counts, BC detection, and specific health biomarkers. All samples will be archived and stored at the University Biobank Limburg (UBiLim). Country-specific protocol variations apply, as ethical and regulatory restrictions prevent blood collection in Denmark and Greece; blood-based biomonitoring data cannot be included in the pooled analysis of the entire three-country cohort. Therefore, analyses based on these specific biomarkers will be limited to the Belgian cohort, and these data will be used to validate external-to-internal exposure pathways for this specific cohort.

#### 3.2.2 Urine sampling

During IP1 and IP2, two urine samples are collected from each participant, the first on Monday morning of Week 1 and the second on Friday afternoon of Week 2 (Figure 7). To directly correlate their external exposure to internal dose, a stratified, data-driven strategy is employed for targeted biomarker analysis. The selection of urinary biomarkers of exposure will be informed by the results of the preceding air samplings. For example, if certain compounds (e.g., benzene or pyrene) are consistently detected or show significant variance during the air sampling campaigns, their corresponding specific biomarkers of exposure (e.g., SPMA for benzene and 1-OHP for pyrene) will be quantified via GC–MS or LC–MS/MS. This allows for precise assessment of children’s internal dose and direct correlation with the measured school environment. Additionally, samples will be screened for general biochemistry and contaminants. Country-specific protocol variations apply, as ethical and regulatory restrictions preclude urine collection in Greece. To address the impact of these biomonitoring variations across cohorts, environmental monitoring will serve as the primary, standardized exposure metric to link with health outcomes (as described in Section 4) for pooled exposure-response analyses of the entire cohort across the three countries. Analyses relying on urinary biomarkers will be stratified and limited to the Belgian and Danish cohorts to further investigate internal exposure pathways and complement environmental data.

## 4 Health outcomes

To investigate the health impact of IAQ and the planned intervention, cognitive performance and blood pressure (BP) are assessed as primary outcomes at multiple time points. Additionally, clinical examinations evaluate secondary outcomes, including psychological well-being, anthropometric measurements, and the skin microbiome. All measurements are performed in an examination room provided by the participating school. The examination room is appropriately lighted and ventilated. A CO₂ measuring device is placed to monitor air quality levels. CO₂ levels are regulated by opening windows and doors at appropriate times. Two testing days are scheduled at BaP. The subsequent testing days occur on the final day of IP1 and the final day of IP2 (Figure 7).

### 4.1 Questionnaires on background information, mental health, and indoor air quality in schools

Four questionnaires are provided to collect general information regarding the lifestyle and living situation, mental health, and well-being of the child. The first of these, focusing on the baseline parameters detailed in Table 3, is to be completed by parents or guardians prior to the start of the BaP.

**Table 3:** Information provided by parents/guardians of the participant in the questionnaire.

Category	Parameters
Personal information	Child full name Class and school Parent email Birthdate Sex
Parental information & socioeconomic status	Respondent relation Country of birth Highest eduction Occupation Financial situation Home ownership status
Demographic/residential information	Current living address(es) (duration) Previous living address(es) (duration) Home heating systems
Environmental exposures	Household smoking status Transport type (time) Exposure to busy traffic Household noise exposure
Health and medical history	General Health Chronic condition Respiratory health Medication/supplement use Allergies
Early life exposures	Birth order Birth weight Birth method Address during pregnancy Complications pregnancy Gestational age Smoking status pregnancy Breastfeeding
Lifestyle and physical activity	Physical activity (type, duration) Indoor/outdoor play time Screen time Sleep duration and quality Eating habits

Two additional questionnaires are completed by the children on the measuring days at the end of IP1 and IP2: the Kidscreen-27 (49) and the Strengths and Difficulties Questionnaire (SDQ) (50) (Figure 7). The Kidscreen-27 is an instrument with 27 items that measure 5 parameters: physical activity and health, feelings and self-image, family and leisure, friends, and school and learning. It takes around 15 min for the children to complete this questionnaire, which is done in a calm environment. The SDQ is a screening list of 25 items that measures 5 parameters: hyperactivity/attention deficit, problems with peers, emotional problems, prosocial behavior, and behavioral problems. The questionnaire also takes 15 min to complete and measures the presence of psychosocial problems, the strengths of the child, and the influence of psychosocial problems on daily functioning.

The final questionnaire is administered on the second measuring day of the BaP, as well as on the testing days of IP1 and IP2 (Figure 7). This questionnaire assesses subjective perceptions of IAQ (SIAQ) in the classroom. Children are asked to evaluate the air quality, report any experienced symptoms, and indicate whether they perceive any thermal discomfort or noise disturbances potentially associated with the study devices or the ventilation system.

The study utilizes both established and project-specific measures: the highly validated Kidscreen-27 (51) and SDQ (52) are available in multiple languages, whereas the baseline questionnaire and SIAQ were tailored specifically for the LEARN project and translated into Greek, Danish, and Dutch by native speakers.

### 4.2 Blood pressure and anthropometric measurements

Since air pollution exposure can affect BP (8, 53), BP is measured at the end of the BaP, as well as on the measuring days of IP1 and IP2 (Figure 7). To account for 24-h BP variability (BPV) (54), all measurements are performed between 08:00 and 12:00 h consistently. Children are asked to rest for 15 min. Afterward, the measurements are conducted on the non-dominant arm. Systolic BP (SBP), diastolic BP (DBP), and heart rate (HR) are noted down. The exact time of each measurement is recorded, along with the initials of the researcher conducting the assessment and possible deviations in the standard operating protocol (SOP). An automated BP instrument (Omron M3 comfort) is used to make five consecutive BP readings (1 min in between) using a pediatric cuff with the correct size. The first two measurements serve to acclimate participants to the environment and minimize stress. The final three readings will be averaged to adjust for any remaining stress-related fluctuations and used for the analysis. Additional measurements might be necessary if errors occur.

Additionally, anthropometric measurements, including height, weight, and waist circumference, are recorded at BaP. Height is measured using the SECA 213 stadiometer. Children are asked to remove their shoes and socks and stand in a freely upright position. Weight is measured using an Omron BF511 body composition monitor. Waist circumference is measured with a Seca 201 anthropometric tape. For this, the child is asked to make their belly button visible. These parameters, which can influence blood pressure, will be accounted for in the analysis (55). Any deviations from the SOP are noted down.

### 4.3 Cognitive assessment

To assess the short-term effects of improved IAQ on cognitive function, a series of cognitive tests is performed on tablet devices under the supervision of trained staff. The program ‘Testmanager Minds’ is applied to conduct the tests (56). This is a software package for administering and scoring automated (neuro)psychological tests. Assessment instruments are provided in Dutch, Danish, and Greek. The Dutch version is validated and drawn from prior studies, while the Danish and Greek versions have been officially translated and approved by native-speaking scientists. All language adaptations were integrated in collaboration with the original program developer to ensure technical and linguistic accuracy. A revised standardized protocol for administering cognitive tests was developed and tested during the pilot. This updated protocol includes the addition of audio recordings, providing standardized test explanations for each participant. The audio recordings are provided using noise-canceling headphones. A secondary purpose of using noise-canceling headphones is to minimize ambient noise and environmental distractions during cognitive testing. Because this protocol is standardized across all subjects, study phases, and sites, the baseline testing conditions remain fully comparable, thereby neutralizing potential confounding effects on concentration and cognitive performance. Additionally, desk dividers are installed between the children to reduce visual distraction.

The cognitive tests are always performed in the following order:

1. Continuous Performance Test: This test measures a child’s sustained attention and concentration using alphanumeric stimuli. A series of letters appears on the screen for 500 ms each. The child must press the spacebar when they see the letter “A” followed by “X.” To ensure understanding, a practice round of 20 letters is given. If the child achieves at least 80% accuracy, they proceed to the main test, which consists of 300 letters. If not, the instructions are repeated, and a new practice round is provided. Key variables recorded include the mean reaction time and amount of correct answers.
2. Symbol Digit Modalities Test: This test assesses the child’s ability to process information. The child is presented with 9 different symbols associated with the numbers 1 to 9. In the default setting, the participant is presented with a number of sequences of symbols for 90 s that have to be combined with the correct numbers. The child is presented with 10 practice symbols before the beginning of the test, in which mistakes are indicated and can be corrected. The participant can manually indicate the response on a virtual keypad. Key variables recorded include total latency and accuracy.
3. Memory Span Task: This test evaluates the child’s short-term memory. It consists of two subtasks for sequences of numbers, both forward and backward, which are presented visually. An ascending sequence of digits is presented that should be reproduced in the same order in the Forward condition, and backward in the Backward condition. Sequences of a given sequence length are offered 2 or 3 times. If two sequences of the same length are reproduced correctly, the sequence length is increased by 1 element; for 1 error, the third sequence is offered as a ‘retake’. At 1 sequence correct out of 3 attempts, the subtask is aborted. Answers are typed in on a virtual keypad. This test does not include a practice round before the actual test begins. Key variables recorded include span length and response latency for both the forward and backward tasks.
4. STROOP Task: This test measures the child’s selective attention. A total of four buttons are shown on the screen (red, blue, yellow, and green), and the names of these colors appear on the screen in the same or a different color than the name indicates. As fast as possible, the children need to press the button that indicates the color the word is shown in, ignoring the color that the name indicates. Before the beginning of the test, a total of 12 practice trials are given, followed by 48 test trials. The real test begins once the child achieves at least 80% accuracy. Key variables recorded include reaction time.
5. Signal Detection Test (SDT): This test evaluates a child’s visual information processing speed. It assesses perceptual sensitivity and response bias through multiple short trials featuring alphanumeric stimuli. The goal is to quickly determine whether a target symbol (U) is present among distractors (V) in a two-choice task. Children respond “yes” if they see a U among the *Vs* and “no” if they do not. The test consists of 60 trials, including a 10-trial practice round. The real test begins once the child achieves at least 50% accuracy. Key variables recorded include sensitivity and response bias. However, in the pilot study, the tablets used for testing often lacked the processing power to run the SDT test software, leading to frequent interruptions or complete data collection failures. These technical issues compromised the test’s reliability and led to significant missing data. Consequently, the SDT was discontinued and will be excluded from the final analyses to ensure the overall robustness of the study’s conclusions.

Children perform the cognitive tests at four time points (Figure 7). During the BaP, each child completes all cognitive tests twice on different days to account for potential learning curve effects (57). Afterward, the children repeat the tests during the intervention and sham-intervention phases on the testing days to evaluate the impact of IAQ. To control for any residual learning effects, the specific intervention period will be included as a covariate in the mixed-model analysis. Cognitive testing is standardly performed between 08:00 and 12:00 h to account for variations in concentration dependent on the time of day (58, 59). The supervisor documents possible distractions and lapses in concentration. Children are given a score based on their attentiveness during the cognitive testing (0 = no problem, 1 = distracted, 2 = influenced by someone, 3 = external distractions, 4 = did not understand the task, 5 = bored, 6 = other). A score is assigned for each of the cognitive tests. Reasoning for the score is also noted down.

### 4.4 Skin microbiome

On the same days as blood collection, between 08:00 and 12:00 h, skin microbiome samples are collected using eNat® swabs (designed for nucleic acid collection and preservation) hydrated with 0.9% NaCl solution (Figure 7). These samples will be stored at UBiLim until further processing and analysis. Country-specific protocol variations apply, as ethical and regulatory restrictions prevent collection in Denmark and Greece.

## 5 Data management and statistical strategy

### 5.1 Data collection methods

All data are collected by trained and certified researchers affiliated with the LEARN project team. Prior to data collection, all personnel involved received comprehensive training to ensure standardization and adherence to established SOPs for environmental and biological sample collection, clinical measurements, and cognitive assessments. After the sampling campaign at each school, each child will receive a certificate of participation, and each participating class will receive a new board game developed within the LEARN project to help children learn about IAQ in schools.

### 5.2 Data management

The samples will be stored for 25 years after completion of the project; blood, urine, and skin swab samples will be stored in freezers at −80 °C in each country. At the end of the collection process, the biological samples are sent to Belgium for analysis and subsequent storage at UBiLim. The research is part of the LEARN project, which started in May 2022 and will be completed in 2026. Afterward, the samples will be securely disposed of, and the data will be transferred to and stored on a secure data hub, which will be established in collaboration with our partners at Envirometrics S. A., so that all data will be available for at least 15 years after the publication of the results or the end of the funding period. Personal data will be stored until the conclusion of the project. Completion of the project is defined as the moment when manuscripts describing the project results are accepted for publication.

### 5.3 Statistical strategy

Descriptive statistics will be used to summarize participant, classroom, and school characteristics; indoor air pollutant levels; microclimate parameters; biomarker concentrations; cognitive performance; and health outcomes.

To evaluate the impact of the intervention (vs. sham-intervention) and IAQ on longitudinal child outcomes, specifically cognitive performance and clinical/biological parameters (e.g., blood pressure), linear mixed-effects models will be applied. This model is specifically chosen to account for the longitudinal nature of the data, as multiple measurements will be collected from the same child. To handle the hierarchical clustering, random intercepts will be specified for school, class, and child. Background variables will be included as fixed-effect covariates in the models. Questionnaire data and clinical/pollution measurements will provide the necessary parameters to adjust for individual (e.g., socioeconomic factors and anthropometric measurements) and environmental (e.g., classroom pollution exposure, season) confounders, selected according to the requirements of each distinct analysis. Data collected from pre-sampling screening questionnaires will be used to adjust for building characteristics (e.g., building age, ventilation system) and external environmental factors (e.g., traffic exposure).

To analyze differences in environmental exposure levels (both external pollutants and internal exposure parameters) across different study phases (intervention vs. sham-intervention) and subgroups (e.g., schools), paired statistical tests (e.g., Student’s t-test, Mann–Whitney U test, or Wilcoxon signed-rank test) and ANOVA (or Kruskal-Wallis test, or Friedman test) will be used. The relationship between various parameters will be statistically analyzed using correlation and regression analysis.

## 6 Discussion

### 6.1 State-of-the-art

While there is growing recognition of the adverse effects of indoor and outdoor air pollution on children’s health, the specific role of the school environment has received comparatively less attention. Several studies have observed the adverse effects of air pollution exposure on cognitive development and cardiovascular health in children, yet limited studies have indicated the importance of air quality in schools to children’s health (6–8, 60, 61).

A prospective study conducted in Spain studied the impact of traffic-related air pollutants on the cognitive development in children aged 7–10 (9). Schools were classified into high- and low-pollution groups based on modeled nitrogen dioxide (NO₂) levels. The findings revealed a slower progression in cognitive development among children attending schools in higher pollution areas, emphasizing the critical need for effective air quality regulations in educational settings. More recently, a randomized, double-blinded crossover study in China (2024) demonstrated the cognitive benefits of indoor air filtration in college students (5). Researchers observed that a significant reduction in indoor air pollutant concentrations was followed by a corresponding increase in student test scores. These results suggest that even a short-term reduction of PM concentrations can significantly improve cognitive function. Given that children are inherently more vulnerable to these effects, the necessity of the present study is further underscored.

The association between PM_2.5_ exposure and BP in children was explored in several studies. In 2022, this association was observed in school children aged 9–10 at elementary schools in Korea (10). Notably, this study assessed both indoor and outdoor PM_2.5_ levels at children’s homes, classrooms, and surrounding outdoor environments. A significant relationship was observed between elevated PM_2.5_ exposure and increased BP, reinforcing the relevance of indoor exposures during school hours. A 2025 randomized crossover trial involving 79 children (average age: 10) in China also demonstrated the significant cardiovascular benefits of air purification (22). By installing air purifiers in both classrooms and bedrooms, researchers compared true intervention against a sham control. The results revealed a significant decrease in SBP alongside a substantial reduction in PM_2.5_ levels. While this study confirms the benefits of air purification in high-pollution environments, the LEARN study seeks to determine if these cardiovascular improvements persist in regions with different levels of ambient air pollution.

**Figure 8:**
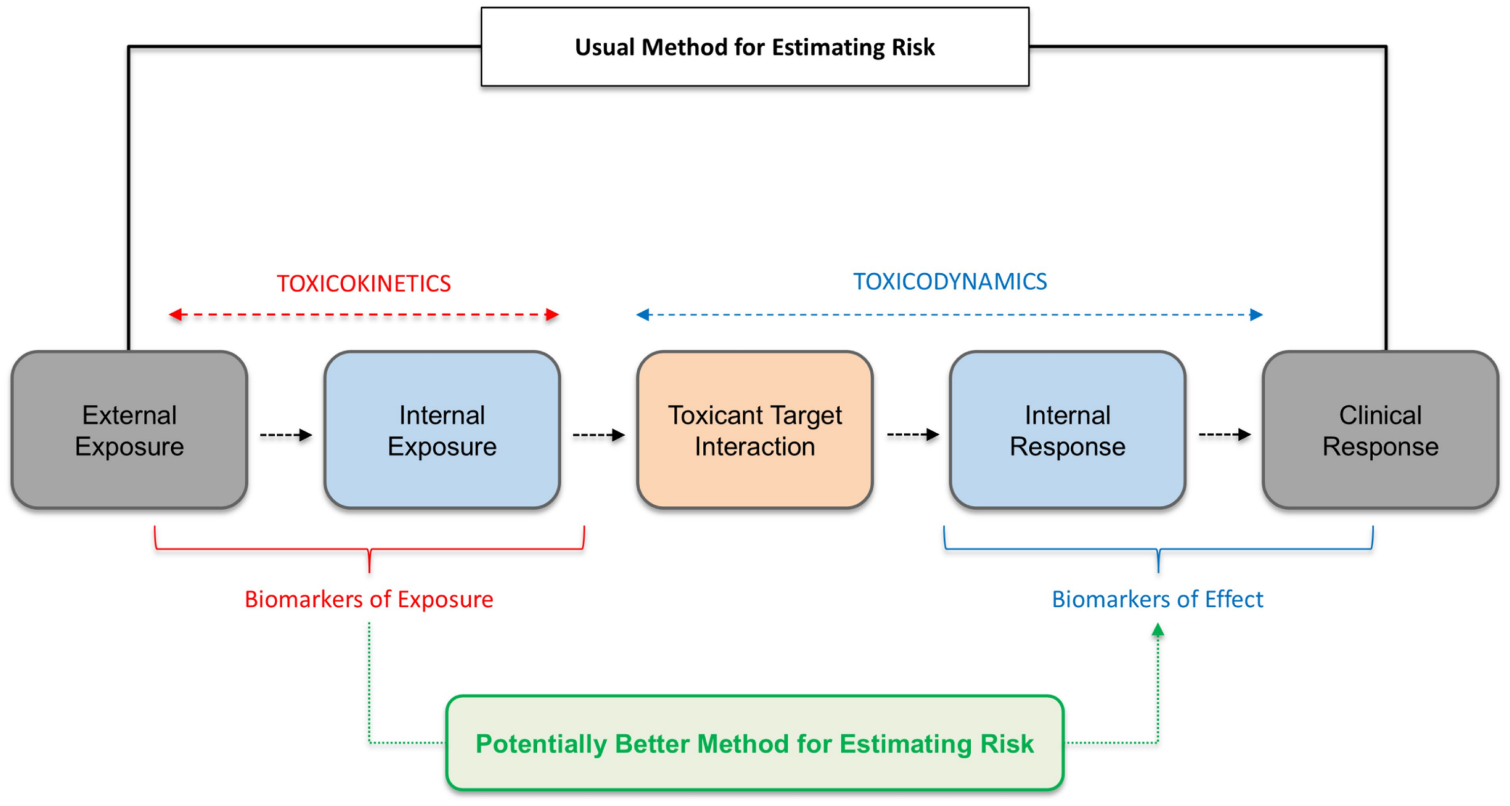
Rationale for using biomarkers to assess risk (adapted from Louro et al.) (64).

Traditionally, environmental monitoring is the primary approach to assess IAQ. However, this approach is costly, time-consuming, and labor-intensive. It reflects potential external exposure only and does not account for factors such as breathing rate and individual variations in susceptibility, which can lead to large uncertainties in extrapolating human health effects from air pollutant measurements (62). In contrast, human biomonitoring has unique advantages and is considered the “gold standard” (63) for assessing human exposure to environmental pollutants. Human biomonitoring is a method for assessing human internal exposure to chemicals by measuring the parent chemicals or their metabolites (namely biomarkers) in human specimens (64). It accounts for the presence of chemicals absorbed by the body through all pathways (e.g., inhalation, dermal, and ingestion) and multiple sources, thereby providing better information on the interrelationships between exposure, dose, and possible health effects (Figure 8) (24, 64).

The LEARN project wants to fill these research gaps through a comprehensive approach that includes the assessment of the air quality in the classrooms, the investigation of internal exposure via biomonitoring strategies, and the implementation of an intervention trial. It is an innovative study to characterize IAQ in classrooms and its impact on children’s health and well-being. As part of the LEARN project, this study develops and implements an integrated approach to understand the relationship between indoor air exposure at school, internal exposure of biomarkers, and cognitive outcomes of schoolchildren, and to introduce and evaluate novel remediation strategies in schools.

While previous research has established that air purifiers can improve children’s cognitive performance and blood pressure in specific settings, it remains to be seen how these benefits translate across varying levels of air pollution. By conducting our research across Belgium, Denmark, and Greece, we specifically account for the regional variations and environmental factors that influence the degree to which students actually benefit from these interventions.

### 6.2 Main strengths and weaknesses

The key strength of this study lies in its design. Comparing health outcomes, internal biomarkers, cognitive function, and well-being with IAQ before and after the application of a novel remediation strategy in a single-blinded crossover design allows for the precise determination of the effectiveness of the intervention. The crossover design also allows the same group of children to serve as their own controls, minimizing variability and providing a better understanding of the intervention’s health benefits. Due to the practical necessity of researchers installing the equipment, a single-blinded design was employed.

Another strength of this study is the carefully designed cognitive testing protocol. The potential for training effects was addressed by including two practice sessions during the BaP (57). Additionally, the use of standardized audio instructions ensures consistent delivery of test explanations across participants. Earlier studies often relied on oral explanations given by test administrators, which can introduce variability and potentially influence the results (65).

The same logic applies to the BP measurements. A baseline measurement is performed to allow children to become familiar with both the procedure and the researchers. During the BP assessments, five consecutive measurements are taken at each time point; the first two serve as internal acclimatization and will be excluded from the final analysis. This approach helps to minimize stress-related responses that can elevate BP readings during initial assessments (66). By addressing the acclimatization effect, the reliability of the blood pressure data is improved.

A third strength of this study is the modified standardized screening questionnaire with a scoring system. These questionnaires are given to and completed by the school staff responsible for each participating class. This enables easy identification of potential sources of indoor air pollutants in participating classrooms and ensures comparability between the two participating classrooms before the sampling campaign.

A fourth strength is the comprehensive environmental measurements, including VOCs and aldehydes, during the “Intervention Periods.” Unlike previous studies that only selected one approach for sampling duration, we perform continuous passive air sampling throughout the school hours, during the whole week (except weekends), in combination with active sampling 3 days per week during school hours. This design allows for the evaluation of overall and daily changes in levels of VOCs and aldehydes during each “Intervention Period,” and their potential relationships with classroom activities, weather, and other parameters. The dual sampling approach also allows us to avoid potential over- or underestimation of VOCs and aldehydes concentrations due to the inclusion of unoccupied periods in the sampling interval, as seen in previous studies (44, 67).

A fifth strength is that two urine samples are collected on Monday morning and Friday afternoon, respectively, to distinguish between school/home exposures. Monday’s samples are collected after being at home over the weekend to reflect primarily home-based exposure, whereas Friday’s samples are collected after 5 days of school (and at home in the evening) to reflect the integrated exposure at home and school. By comparing biomarker levels between the samples from these two time points, the effects of school-specific exposures and interventions can be more accurately assessed, minimizing confounding by home or out-of-school exposures.

On the other hand, there are also some limitations. A notable limitation is the exclusion of children and/or guardians with insufficient local language proficiency, which was logistically necessary to ensure the accurate completion of questionnaires and cognitive assessments. However, this criterion may introduce selection bias and limit the overall representativeness of the study population. Specifically, it potentially underrepresents certain vulnerable groups, such as children from recently immigrated families.

Additionally, although standardized procedures are implemented across all participating countries, some environmental monitoring strategies and measurements vary between sites due to local resource constraints or ethical limitations. This heterogeneity across study sites represents a limitation that could affect data comparability. To minimize this bias, we established strict standardized guidelines for data harmonization and will utilize appropriate statistical modeling to mathematically account for and evaluate between-country differences.

Furthermore, although children spend a significant portion of their time in classrooms, they are also exposed to various pollutants outside of school hours. While we estimate their overall exposure based on their home address, we are unable to account for time spent at other locations, such as during extracurricular activities or hobbies. We also could not account for traffic-related pollution during school commutes.

A final limitation of this study is the high participant burden associated with the intensive testing regimen and repeated cognitive sessions, which could introduce fatigue or boredom effects. However, this risk is mitigated by our highly structured scheduling designed to minimize missed school hours, the expertise of our pediatric-focused research team in keeping children comfortable, and strict adherence to participant autonomy, allowing children to opt out of tests at any point.

## 7 Conclusion

Here, we present a single-blinded crossover study protocol to investigate children’s indoor air exposure in primary schools in combination with an intervention of a novel air remediation strategy. The study will be conducted in Belgium, Denmark, and Greece using harmonized, standardized procedures. This approach measures and characterizes the levels and profiles of indoor air pollutants and microclimate parameters in schools, combined with the analysis of biomarkers of exposure, health parameters, and cognitive testing in children, to provide a comprehensive understanding of indoor air pollution in schools and its effects on children’s cognition, health, and well-being.

## Glossary

ANOVA: Analysis of Variance
BC: Black Carbon
BaP: Baseline Period
BP: Blood Pressure
BPV: Blood Pressure Variability
CO₂: Carbon Dioxide
DBP: Diastolic Blood Pressure
EU: European Union
FID: Flame Ionization Detector
GC: Gas Chromatography
GIS: Geographical Information Systems
HPLC: High-Performance Liquid Chromatography
HR: Heart Rate
IAQ: Indoor Air Quality
IP1: Intervention Period 1
IP2: Intervention Period 2
MS: Mass Spectrometry
NO₂: Nitrogen Dioxide
PAH: Polycyclic Aromatic Hydrocarbons
PM: Particulate Matter
PTFE: Polytetrafluoroethylene
SBP: Systolic Blood Pressure
SDT: Signal Detection Test
SOP: Standard Operating Protocol
SVOCs: Semi-Volatile Organic Compounds
UFP: Ultrafine Particles
UV: Ultraviolet
VOCs: Volatile Organic Compounds
WBGT: Wet Bulb Globe Temperature
WHO: World Health Organization
WP: Washout Period
